# Long survivor family with HIV infection. Case presentation

**DOI:** 10.1186/1471-2334-13-S1-P56

**Published:** 2013-12-16

**Authors:** Alina Cristina Neguț, Anca Streinu-Cercel, Oana Streinu-Cercel, Mihai Lazăr, Adrian Streinu-Cercel

**Affiliations:** 1 Carol Davila University of Medicine and Pharmacy, Bucharest, Romania; 2 National Institute for Infectious Diseases “Prof. Dr. Matei Balş”, Bucharest, Romania

## Background

After the advent of highly active antiretroviral therapy, HIV infection has turned into a chronic disease and the patient’s profile has changed dramatically. We are now treating patients that are active both socially and professionally, in whom virological response has already been achieved and quality of life has become of utmost importance, directly correlated with the management of drug-associated adverse reactions. To describe this new patient profile, we present the management of a young family, living with HIV for more than 7 years.

## Case report

A family was referred to our clinic in the summer of 2012, by the Italian HIV Clinic where they had been under medical evaluation for over 7 years. They had tested positive at the routine pregnancy test 7 years back, when the mother was started on lopinavir/r, lamivudine+zidovudine. She gave birth to a healthy HIV-negative girl, who received postpartum prophylaxis for 3 months (as per Italian protocol). After delivery the mother was switched to efavirenz plus lamivudine+zidovudine, the same therapy as her husband’s, a regimen they would both take for 7 years.

In 2012 they moved to Romania and started attending our clinic. Both had undetectable viral loads and high CD4 counts (>700 cells/cmm), consistent with their medical history. However, both complained of lipodystrophy. The female also complained of peripheral neuropathy, sleep disturbance, vivid nightmares while the male presented increased serum cholesterol and triglycerides, irritability and sleep disturbance.

Because their quality of life had started to decrease, after psychological evaluation and support, therapy was changed to raltegravir, abacavir+lamivudine (both had negative HLA-B*5701). This regimen was well tolerated, the symptoms improved, the CD4 count remained high, the male’s lipid profile returned to normal, and dual-energy X-ray absorptiometry (DEXA) evaluation at 3 months showed an almost normal lipid distribution for both patients, with a slight tendency toward android abdominal lipid distribution for the female patient. She remained undetectable (data available for 1 year follow-up), however, in the case of the male patient HIV-RNA rose to 320 copies/mL at 3 months, possibly associated with the impact of the drug regimen on viral reservoirs. At 6 months, the viral load became undetectable and remained that way to date.

## Conclusion

Correct and prompt management of adverse reactions is an important aspect in patients on antiretroviral therapy, since this may lead to improved quality of life and better social and professional integration of patients.

